# Pan-Cancer and Single-Cell Modeling of Genomic Alterations Through Gene Expression

**DOI:** 10.3389/fgene.2019.00671

**Published:** 2019-07-18

**Authors:** Daniele Mercatelli, Forest Ray, Federico M. Giorgi

**Affiliations:** ^1^Department of Pharmacy and Biotechnology, University of Bologna, Bologna, Italy; ^2^Department of Systems Biology, Columbia University Medical Center, New York, NY, United States

**Keywords:** NGS (next generation sequencing), genomics, cancer, TCGA, single-cell sequencing

## Abstract

Cancer is a disease often characterized by the presence of multiple genomic alterations, which trigger altered transcriptional patterns and gene expression, which in turn sustain the processes of tumorigenesis, tumor progression, and tumor maintenance. The links between genomic alterations and gene expression profiles can be utilized as the basis to build specific molecular tumorigenic relationships. In this study, we perform pan-cancer predictions of the presence of single somatic mutations and copy number variations using machine learning approaches on gene expression profiles. We show that gene expression can be used to predict genomic alterations in every tumor type, where some alterations are more predictable than others. We propose gene aggregation as a tool to improve the accuracy of alteration prediction models from gene expression profiles. Ultimately, we show how this principle can be beneficial in intrinsically noisy datasets, such as those based on single-cell sequencing.

## Introduction

Cancer is a molecular disease occurring when a cell or group of cells acquire uncontrolled proliferative behavior, conferred by a multitude of deregulations in specific pathways (Hanahan and Weinberg, 2011). As is implied by such a broad definition, cancer is a highly heterogeneous disease, showing remarkably different molecular, histological, genetic, and clinical properties, even when comparing tumors originating from the same tissue (Meacham and Morrison, 2013). Many cancers are characterized by the presence of single nucleotide or short indel mutations and/or copy number alterations, which appear somatically at the early stages of oncogenesis and can drive tumor progression (Bozic et al., 2010). Cancers can be broadly divided in two classes: the M class, where point mutations are prevalent, and the C class, where copy number variations (CNVs) are more numerous and are often associated with TP53 mutations. Tumor class influences anatomic location. Most ovarian cancers, for example, belong to the C class, while most colorectal cancers belong to the M class, although many exceptions do exist (Ciriello et al., 2013).

The Cancer Genome Atlas (TCGA) project (Chang et al., 2013) has recently undergone a major effort to collect vast amounts of information on thousands of distinct tumor samples. The TCGA data collection, commonly referred to as the “pan-cancer” dataset, provided the scientific community with an avalanche of data on DNA alterations, gene expression, methylation status, and protein abundances among others, with the critical mass necessary to identify rarer driver tumorigenesis effects in many types of cancers (Brennan et al., 2013; Cancer Genome Atlas Network, 2015; Leiserson et al., 2015). By combining all 33 TCGA datasets, Bailey and colleagues (Bailey et al., 2018) recently outlined a pan-cancer map of which mutations can be drivers for the progression of cancer.

The availability of thousands of samples measuring many different variables in cancer has allowed scientists to generate statistical models of relationships between different molecular species. A pan-cancer correlation network between coding genes and long noncoding RNAs, for example, sheds light on the function of non-coding parts of the transcriptome (Liu and Zhao, 2016). More recently, mutations on transcription factors (TFs) have been linked to altered gene expressions and phosphoprotein levels in 12 TCGA tumor type datasets (Osmanbeyoglu et al., 2017). Network approaches have been applied to identify clusters of coexpressed genes, shared by multiple cancer types (Kim and Kim, 2018). Several studies have sought to characterize the relationships between genomic status and expression levels in cancer, trying to identify commonalities across different cancer types (Ghazanfar and Yang, 2016; Sharma et al., 2018). In particular, Alvarez and colleagues (Alvarez et al., 2016) have postulated that the effect of genomic alterations in cancer can be more readily assessed by aggregating gene expression profiles into transcriptional networks, rather than by profiles taken separately.

While the association between genomic events and gene expression is proven in several scenarios, it remains to be seen if it can be assessed in scenarios where fully quantitative readouts are unavailable, such as low-coverage samples. One of these scenarios is single-cell sequencing (Nawy, 2013), often carried out in experiments where thousands of mutations are generated *via* a system of pooled CRISPR-Cas9 knockouts (Datlinger et al., 2017).

To our knowledge, there is no study trying to identify relationships between all genomic alteration events (somatic mutations/indels and CNVs) and global gene expression across cancers. In this study, we use 24 TCGA tumor datasets to investigate whether gene expression can be used to predict the presence of specific genomic alterations in several cancer tissue contexts. To this end, we leverage the current availability of a vast family of machine learning algorithms (Kuhn, 2008). We investigate whether some gene alterations can be better modeled than others and whether using grouped gene expression profiles as aggregated variables can effectively identify specific genomic alterations. Finally, we test whether predicting mutations and CNVs can be carried out in an intrinsically noisy single-cell RNA-Seq (scRNA-Seq) transcriptomics datasets.

## Results

### Collection of Pan-Cancer Dataset

**Figure 1:**
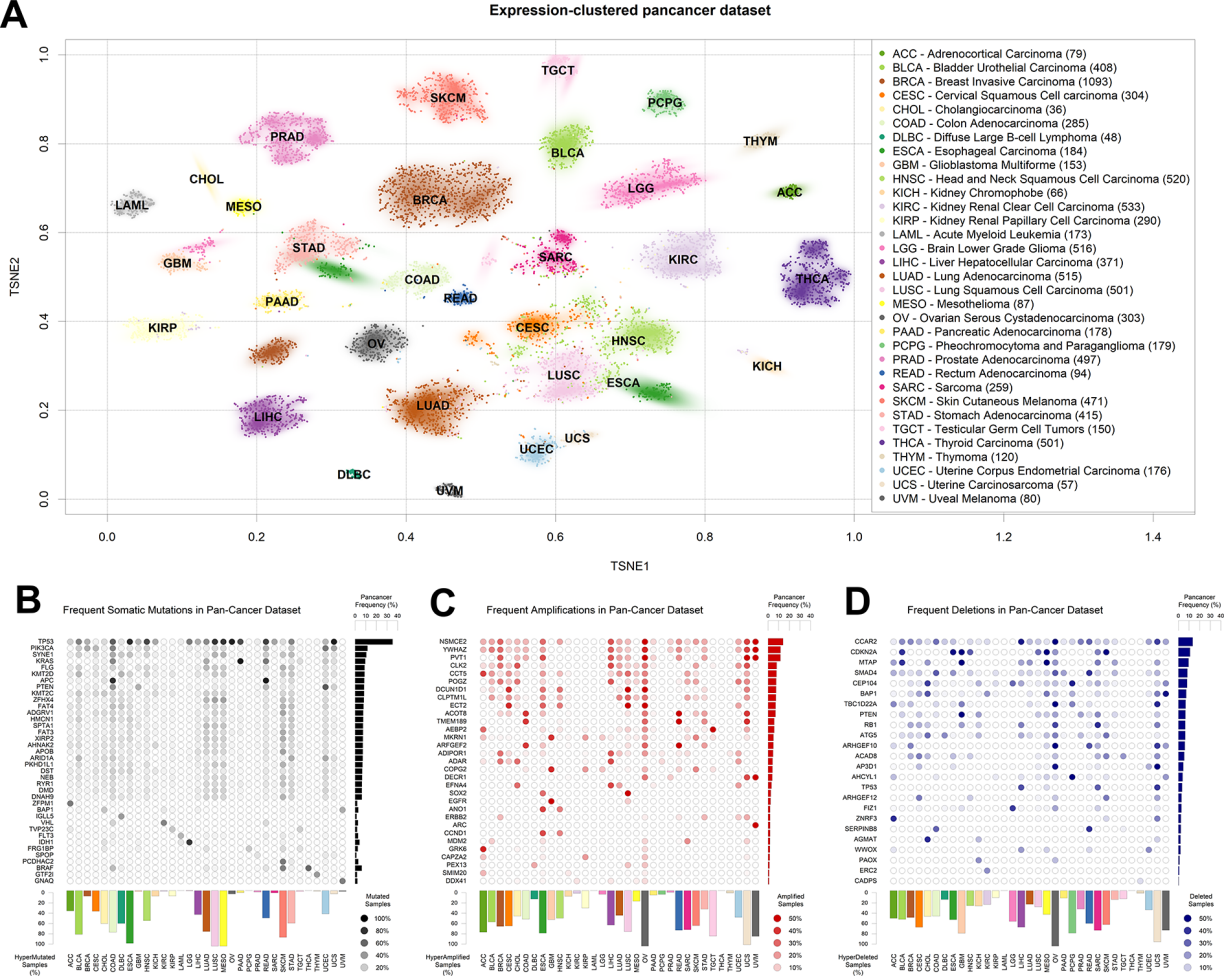
The Cancer Genome Atlas (TCGA) dataset used. **(A)** T-distributed stochastic neighbor embedding (TSNE) clustering of TCGA samples based on gene expression profiles. The 2D median of each tumor type is indicated using the TCGA tumor code. Subset size is indicated in brackets next to tumor type names to the right. **(B)** Table of most somatically mutated genes across TCGA tumor samples, in terms of number of samples where the gene is somatically mutated with altered protein product sequence. **(C)** Table of most amplified genes across TCGA tumor samples. **(D)** Table of most deleted genes across TCGA tumor samples. The fraction of total TCGA samples carrying a gene-targeting event is indicated to the right of panels **(B–D)**, and the fraction of samples where more than 0.5% of the genes is affected by the panel event type is indicated to the bottom of panels **(B–D)**.

We downloaded the most recent version of the TCGA datasets available on Firehose (v2016_01_28), encompassing mutational, CNV, and gene expression data. Initially, we organized the expression data as a matrix of 9,642 samples and 20,531 genes, visualized in **Figure 1A** using T-distributed stochastic neighbor embedding (TSNE; van der Maaten and Hinton, 2008) clustering and two-dimensional (2D)-density estimates for each tumor type. As observed before (Chen et al., 2018), the transcriptional properties of TCGA tumors separate tumor types by tissue of origin. In particular, two tumor types segregate into two subgroups: breast cancer, which subdivides into a major luminal cluster and a smaller (in terms of samples collected) basal cluster (Perou et al., 2000); and esophageal carcinoma, which roughly subdivides into adenocarcinomas and squamous cell carcinomas (TCGA network, 2017).

We then aggregated the single nucleotide and short indel somatic mutation data from the same samples for which we had collected gene expression. As is widely known, TP53 is the most mutated gene in human cancer (**Figure 1B**), followed by PIK3CA, SYNE1, and KRAS. As shown before (Ciriello et al., 2013), some tumor types are characterized by a high presence of somatic mutations. In particular, lung squamous carcinoma (LUSC), mesothelioma, and esophageal cancer carry at least one of these events in almost 100% of the samples in the TCGA dataset. In the figure, we filtered out commonly known nondriver mutations (Lawrence et al., 2013), such as those happening in long genes like TTN and OBSCN, but we kept them in all following analyses for the sake of completion. A representation of all mutated genes, including blacklisted ones, is available in **Figure S1**. Some tumors are characterized by the prevalence of a mutation in a specific gene, such as the G-protein coding BRAF in thyroid carcinoma (Kimura et al., 2003) or IDH1, translating into isocitrate dehydrogenase, in low-grade glioma (Yan et al., 2009).

Finally, we obtained readouts of CNV status for all TCGA samples. CNVs can have different extensions in terms of nucleotides affected and can sometimes encompass entire chromosomes (Shlien and Malkin, 2009) and the thousands of genes therein. In order to limit the number of variables to a more meaningful subset, we assigned a CNV score to every gene, according to the copy number score of the genomic region most overlapping with the University of California, Santa Cruz-annotated gene boundaries (genome version hg19). We then tested models for all genes affected by a CNV in at least 10 samples [extending what was previously done in Chen et al. (2014)]. In order to make CNV variables comparable with the mutational ones, we defined a cutoff for presence or absence by using the log_2_(CNV) threshold of 0.5, which roughly corresponds to at least one copy gain for amplifications, and at least one copy loss for deletions (see Materials and Methods). We then reported their abundance in the pan-cancer dataset, distinguishing between amplifications (**Figure 1C**) and deletions (**Figure 1D**). As previously shown (Ciriello et al., 2013), virtually all ovarian cancer samples are characterized by at least one CNV event. Among the most amplified genes, we find the oncogenes SOX2 (Bass et al., 2009), EGFR (Bell et al., 2005), and MDM2 (Momand et al., 1998), and also a noncoding gene, PVT1, the most amplified gene in breast cancer, with proven but as-of-yet uncharacterized proto-oncogenic effects (Colombo et al., 2015; Li et al., 2017). Among the most deleted genes (**Figure 1D**), we observe well-known tumor-suppressor genes, such as CDKN2A (Usvasalo et al., 2008; Mistry et al., 2015) and PTEN (Zhao et al., 2017; Wang et al., 2018).

### Modeling Cancer Alterations With Gene Expression

After collecting all the expression and genomic alteration data from TCGA, we set out to generate models that are able to predict the presence or absence of each event by virtue of gene expression data in the contexts of all collected tumor types.

**Figure 2:**
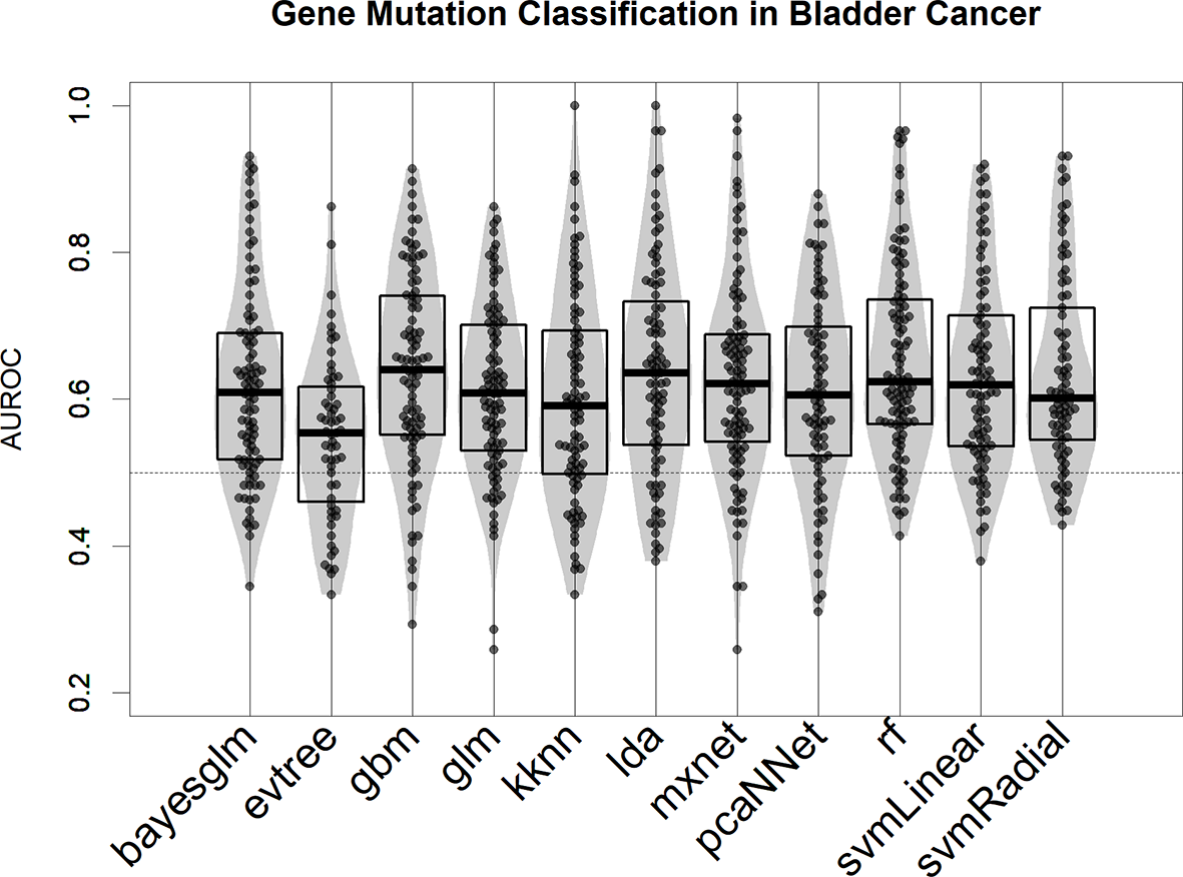
Performance of 11 machine learning algorithms in binary classification of mutated/nonmutated samples using gene expression predictor variables in the bladder cancer dataset. Each point corresponds to a specific mutation/model. Performance is indicated as AUROC: area under the receiver operating characteristic curve.

We tested several modeling algorithms for classification using the aggregator platform for machine learning caret (Kuhn, 2008) in the bladder cancer mutational dataset (Robertson et al., 2017). In our rationale, we tested at least one algorithm from every major machine learning family (decision trees, support vector machine, neural networks, and linear models; see Methods for a full list). We observed that all models provide better-than-random predictions for the majority of mutational events, in terms of area under the ROC curve (AUROC) (**Figure 2**) (Fawcett, 2006). For the bulk of the subsequent analysis, we selected the top-scoring algorithm in this test, the gradient boost modeling algorithm (gbm), a well-established tree-based boosting model (Friedman, 2001), due to its robustness and speed of implementation. In all our test runs (**Figure 2** for bladder cancer and Figure S2 for liver hepatocellular carcinoma), gbm models are not significantly different (in terms of AUROC comparison, two-tailed Wilcoxon Test *p* > 0.1) from other well-performing algorithms, such as linear discriminant analysis or support vector machine.

**Figure 3:**
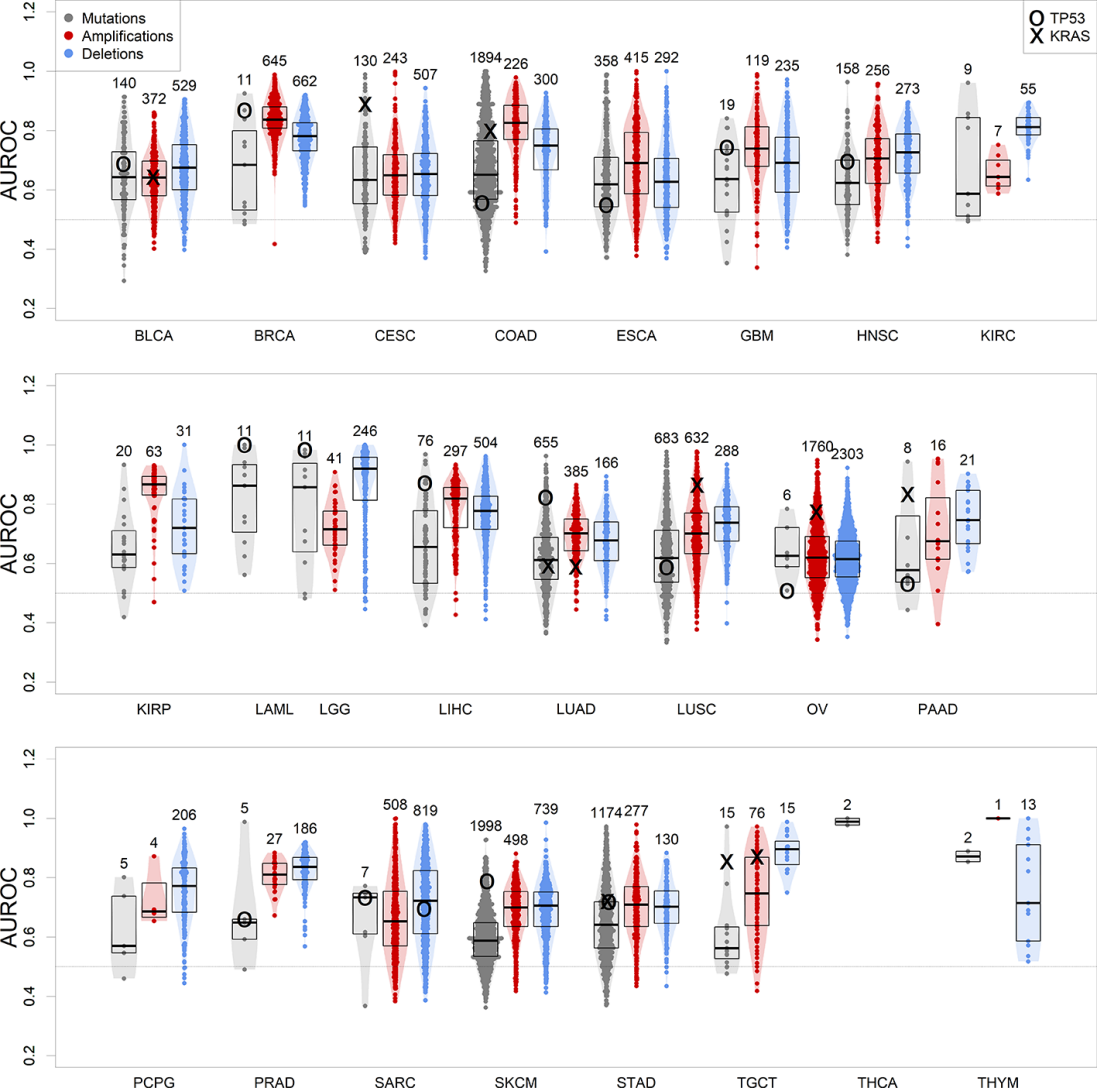
Performance of gbm models for each genomic alteration event in TCGA, predicted as a function of each tumor gene expression. Boxplots indicate distribution median, upper and lower quartile. Alterations targeting TP53 and KRAS are indicated. Numbers on top of the violin plots indicate the number of models generated.

We therefore calculated gbm models for all tumor types of at least 100 samples with co-measured expression and CNV or mutations, which included 24 of the 33 TCGA tumor types. The models were predictive of genomic events observed in no less than 5% and no more than 95% of the patients in the dataset, and at least in 10 samples. Our results show that in all tumor types, a machine learning algorithm based on gene expression is consistently better than a random predictor (AUROC line at 0.5) at correctly classifying tumor samples for the presence or absence of specific genomic alteration events (**Figure 3** and **Supplementary Table S1**).

We focused on TP53 somatic alteration models not only because this tumor suppressor gene is frequently mutated or lost in cancer (**Figure 1**) but also because its loss of function is one of the most common driver events associated to tumorigenesis (Petitjean et al., 2007). In our study, TP53 mutations are well modeled in many of these tumor types (**Figure 3**), being the most well-predicted mutational event in both acute myeloid leukemia and low-grade glioma. In these tumors, loss-of-function somatic mutations of TP53 have been recurrently found as driver events for tumor initiation (Venneti and Huse, 2015; Metzeler et al., 2016 ). We could also model the presence of a copy loss of TP53 in sarcoma, which can be predicted with an accuracy of 70%. Ovarian and pancreatic cancer datasets presented exceptional cases, where TP53 is mutated virtually in all patients (next to 95%) (Cole et al., 2016; Cicenas et al., 2017). This presents a challenge for the modeling algorithm, as there are not enough wild-type samples to perform a robust training (TP53 model performances in these tumors are close to 0.5, i.e. randomness).

We further focused on models predicting KRAS, a very important oncogene whose protein product is fundamental in transmitting proliferation signals in the early steps of the mitogen-activated protein kinase cascade (Tsuchida et al., 1982). KRAS’s role in cancer is caused by specific point mutations in its guanosine triphosphate-binding domain, which make it constantly active and therefore a deregulated signal transducer for proto-oncogenic pathways (Kranenburg, 2005). Our results confirm the key role of KRAS-targeting somatic mutations, which are well modeled by gene expression in KRAS-driven tumors: colon, lung, pancreas, stomach, and testicular cancers, as well as cervical squamous carcinoma (Prior et al., 2012) (**Figure 3**). Less commonly, the oncogenic activity of KRAS can be increased by amplification in ovarian cancer (Huang et al., 2012) and LUSC (Wagner et al., 2011). Our results show that patients can be well separated between KRAS-amplified and KRAS-normal using gene expression in these two tumor types, confirming the presence of a transcriptionally defined subset of patients with KRAS copy number gains.

In general, the observed high variability between somatic mutations and CNVs roots is due to the fact that not all genomic alterations are disease drivers, and some are simply passenger events (Bozic et al., 2010), located either close to the amplified oncogene/deleted tumor suppressor gene, or hypermutated due to deficits in the DNA damage repair mechanisms (Chae et al., 2016), such as the case of skin melanoma (Guan et al., 2015). Differences between mutation and CNV model performances in individual cancer types may be due to the specific characteristics of these. For example, LUSC initiation and progression tend to depend on copy number alterations (Ciriello et al., 2013) rather than somatic mutations, which is highlighted by the highest performance of CNV-predicting transcription-based models over mutation-predicting ones (**Figure 3**). However, the biological heterogeneity observed within cancer datasets does not allow for perfect generalizations, such as tumor types driven exclusively by CNVs or mutations (Smith and Sheltzer, 2018).

We noted a tendency where models for more frequent CNV events yielded a greater predictive power (**Figure S3**), a tendency not observed for somatic mutation models. We then tested if known tumor-related genes, such as those curated by the Cancer Gene Census (Futreal et al., 2004) are better modeled than the rest of the genome. There is no difference in mutation and amplification results, but for deletion events, oncogenes yield weaker models (Wilcoxon test, *p* = 0.0037, **Figure S4**), and tumor suppressor genes yield generally stronger models (*p* = 0.00050). This is in agreement with the central paradigm of cancer, where a tumor suppressor gene deletion can be one of the driving events of tumorigenesis and tumor progression (Sager, 1989). On the other hand, deletion of tumor-promoting oncogenes is generally unfavorable for tumor progression, and so, generally speaking, it should be present only as a passenger event, unlikely to determine global gene expression and tumor fate.

### Modeling Specific Alterations With Noise Addition

In order to understand whether cancer-related genomic alterations can be modeled by gene expression in scenarios with lower signal-to-noise ratio, we artificially perturbed the TCGA gene expression dataset *via* the addition of Gaussian noise and then proceeded to build models to predict the presence of TP53 mutations in breast cancer, the largest dataset in TCGA by number of samples.

**Figure 4:**
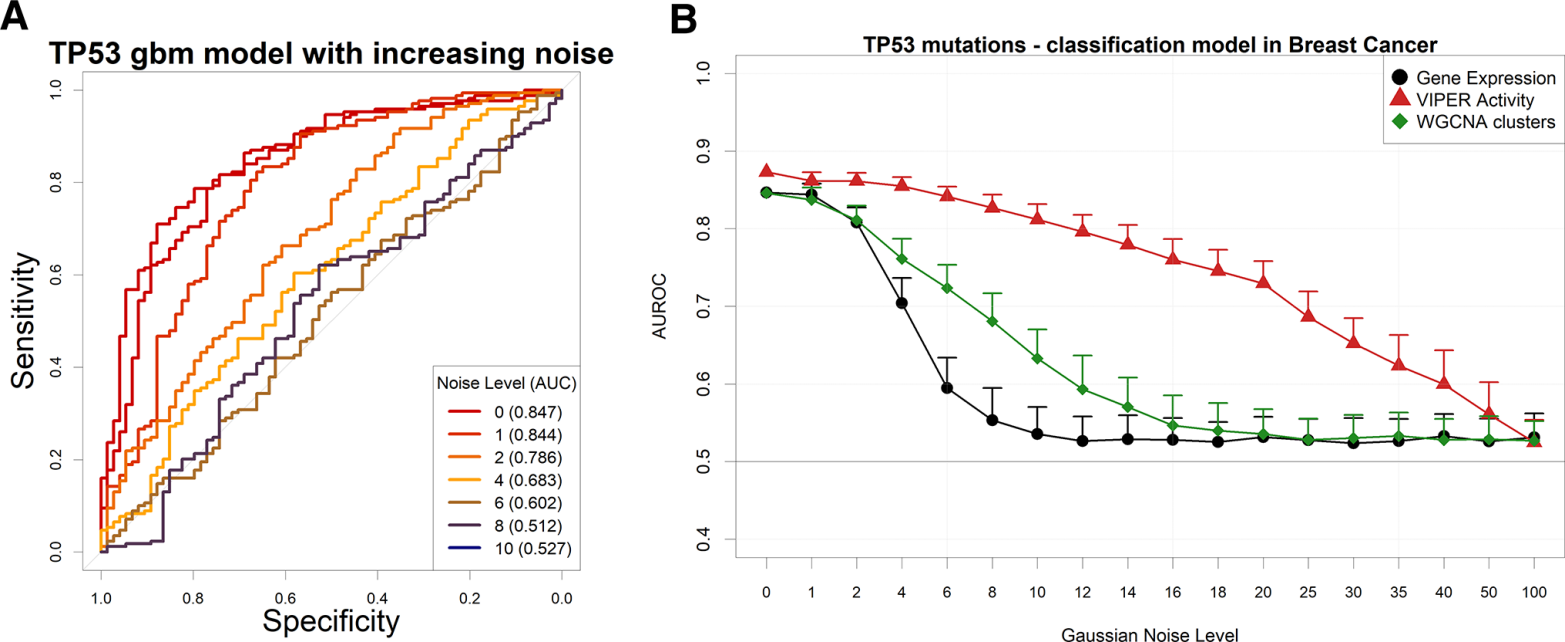
Performance of a TP53 somatic mutation gbm model upon Gaussian noise addiction. **(A)** Receiver operating characteristic (ROC) curves (and area under the curve) upon addition of increasing levels (in terms of SD of a Gaussian distribution with mean = 0) of Gaussian noise. **(B)** AUROCs of the model with increasing noise, calculated using gene expression (black line) or aggregated gene expression using the WGCNA (green line) or VIPER (red line) algorithms. Error bars indicate the standard deviation of AUROC distribution.

As expected, the addition of uniform random Gaussian noise to the gene expression matrix has a detrimental effect on the amount of information left for modeling the presence of TP53 somatic mutations (**Figure 4A**).

We then decided to test several permutations of noise addition on the same breast cancer expression data, by each time aggregating genes into networks defined *a priori* in the same context, using a Tukey biweight robust average method (Irizarry et al., 2006) on weighted gene correlation network analysis (WGCNA) clusters (Langfelder and Horvath, 2008) and the VIPER algorithm (Alvarez et al., 2016) on ARACNe-AP networks (Lachmann et al., 2016). It is important to note that WGCNA clusters are completely nonoverlapping and yield generally a lower number of aggregated variables than VIPER clusters, which are groups of genes possibly shared by other TF clusters and that collectively yield the global expression of a TF target set (dubbed as a proxy for “TF activity” in the original VIPER manuscript; Alvarez et al., 2016).

Our results show that gene expression, VIPER activity, and WGCNA clusters yield very similar models for predicting TP53 mutations in breast cancer (**Figure S5**). The amount of information contained in the input variables is therefore comparable. Adding noise to the input expression matrix, however, and then aggregating the resulting noise-burdened genes into VIPER or WGCNA clusters (see Materials and Methods), provides robustness to the models (**Figure 4B**). Similar results with higher variances (possibly due to the smaller size of the datasets) can be observed for EGFR amplifications in glioblastoma (**Figure S6**) and LUSC (**Figure S7**), for PVT1 amplifications in ovarian cancer (**Figure S8**) and for PTEN deletions in sarcoma (**Figure S9**). In all these examples, however, the performance of the simple WGCNA/Tukey aggregation is closer (if not worse) to that of simple gene expression.

**Figure 5:**
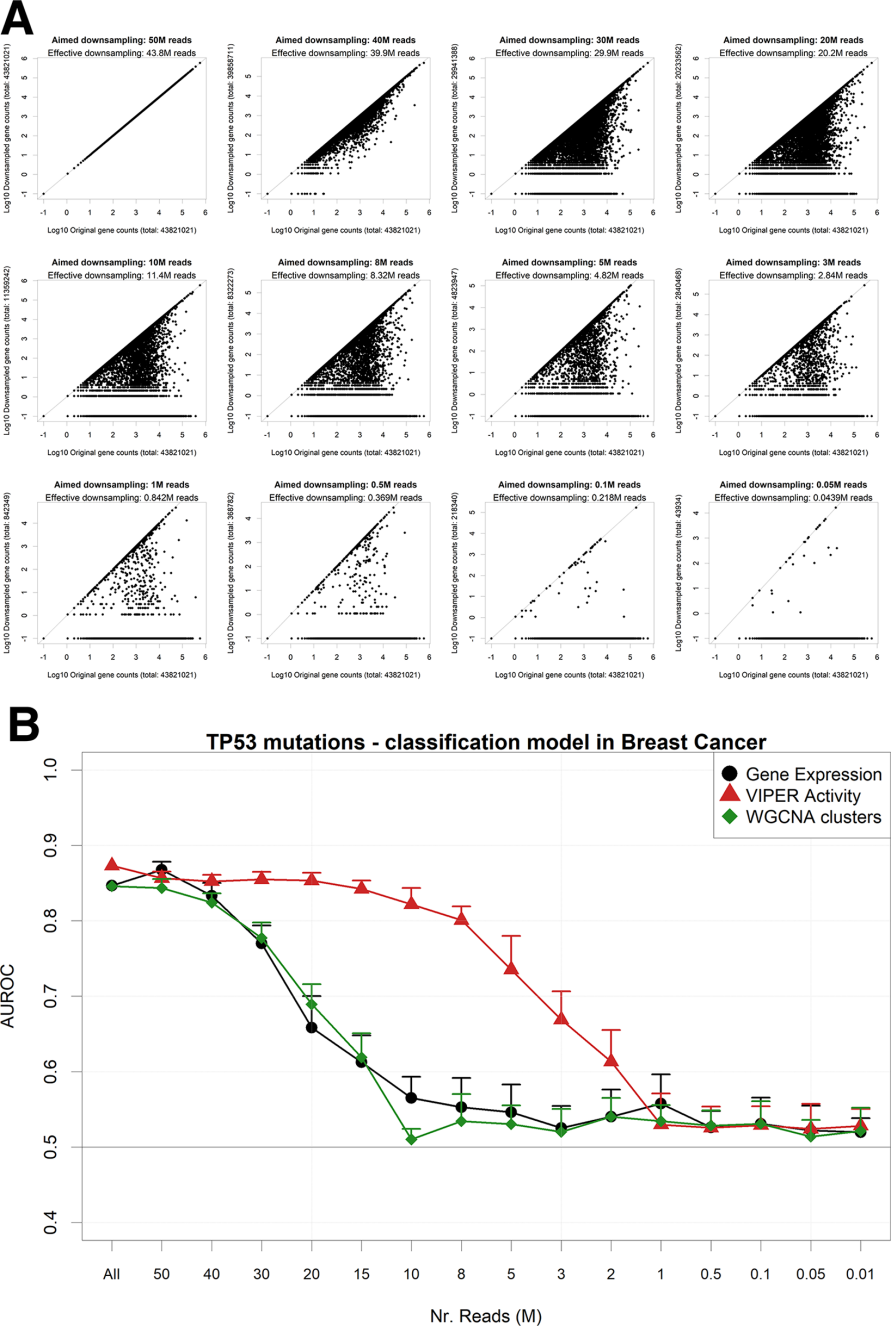
Performance of a TP53 mutation gbm model upon down-sampling of the TCGA breast cancer RNA-Seq dataset. **(A)** for a single TCGA sample (TCGA-A1-A0SB-01) with 43.8 gene mapping reads, the down-sampling algorithm is applied for multiple target read quantities. X-axis shows the count for each gene in the original sample and Y-axis in the down-sampled output. **(B)** AUROCs of the model with decreasing read numbers, calculated using gene expression (black line) or aggregated gene expression using the WGCNA (green line) or VIPER (red line) algorithms. Error bars indicate the standard deviation of AUROC distribution. Pseudocounts of 0.1 are added in order to show zero counts as −1 in log10 scale.

An alternative way to reduce the information content from an NGS gene expression dataset is to reduce the number of read counts from each sample. This operation reflects either a low-coverage bulk RNA-Seq experiment or an experiment arising from single-cell sequencing (Pollen et al., 2014). In particular, single-cell RNA-Seq (scRNA-Seq) is characterized by the dropout phenomenon (Risso et al., 2018) wherein genes expressed in the cells are sometimes not detected at all. In order to simulate such scenarios, we down-sampled each RNA-Seq gene count profile from the largest TCGA dataset (breast cancer) to a target aligned read number using a beta function, which allows for reduction coupled with random complete gene dropouts (**Figure 5A**). We then modeled again the presence of TP53 mutations using gene expression (**Figure 5B**). We found out that models based on standard unaggregated gene expression experience an accuracy drop at around 30M reads, while aggregating genes using VIPER (but not with WGCNA) allows for better-than-random accuracies even at 3M reads, confirming the benefits of gene aggregation in low-coverage RNA-Seq, as previously found e.g. for sample clustering (Bush et al., 2017).

### Mutation Prediction in Single-Cell Data

**Figure 6:**
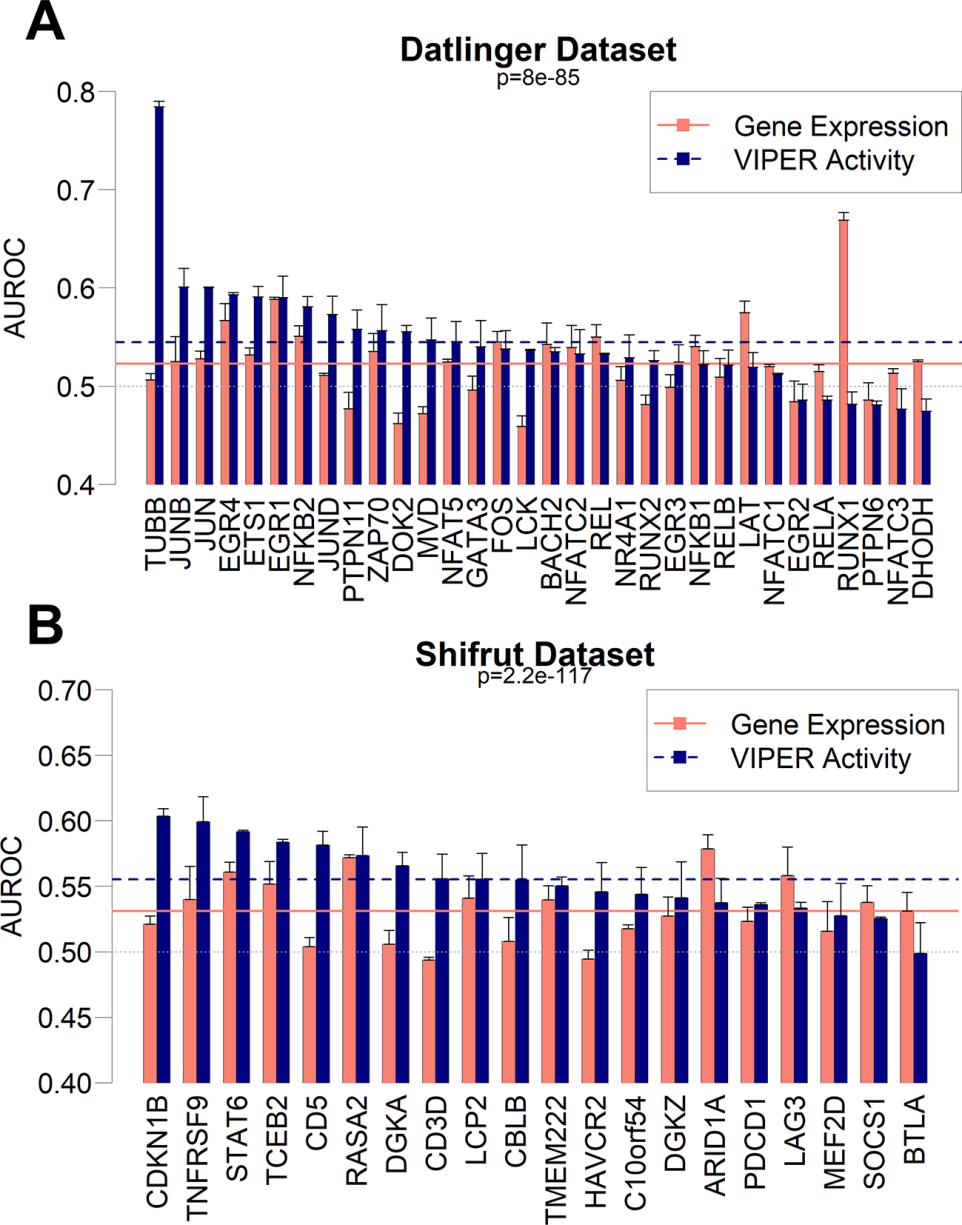
Performance as AUROC of gbm models to predict mutations in CROP-Seq datasets using gene expression (red bars) and VIPER activity (blue bars) derived from CCLE expression data in Datlinger **(A)** and Shifrut **(B)** datasets. The p-value of paired Wilcoxon tests between all VIPER and expression AUROCs in each dataset is reported, as well as the average of all expression models (red solid line) and all VIPER activity models (blue dashed line). Error bars report the standard deviation of 100 AUROCs generated from multiple partitioning of training/test sets. Error bars indicate the standard deviation of AUROC distribution.

Based on the results from the pan-cancer analysis, where we predicted sample mutations based on pooled RNA-Seq gene expression patterns, we decided to extend the same approach on single-cell datasets. Recently, the CROP-Seq methodology has been introduced (Datlinger et al., 2017), allowing for the measurement of cell-specific transcriptome-wide gene expression and mutations induced by CRISPR-Cas9 (Ran et al., 2013), thanks to the concurrent sequencing of CRISPR-Cas9 guide RNAs. We therefore tested the capability of gbm models to predict mutations using gene expression variables in two independent single-cell datasets. The first dataset (dubbed “Datlinger”) was extracted from the Jurkat cell line derived from human T lymphocytes (Datlinger et al., 2017). The second one (dubbed “Shifrut”) derived from primary unstimulated T cells from a human donor (Shifrut et al., 2018). We removed cell unique molecular identifier counts and cell cycle as common confounding effects of single-cell datasets (Tirosh et al., 2016) (**Figure S11**). We generated a regulatory transcription network using ARACNe-AP on the RNA-Seq Cancer Cell Line Encyclopedia dataset (CCLE; Barretina et al., 2012), which comprises 1,021 distinct human cell lines. Using the CCLE network, we aggregated gene expression from the single-cell datasets using the VIPER algorithm and implemented the resulting TF-centered VIPER activity profiles to build prediction models for the Crop-Seq-detected mutations. Parallelly, we built models using un-aggregated variance stabilizing transformation (vst)-normalized gene expression data. Our results show that gbm models based on VIPER activity variables globally achieve a significantly higher performance in both the Datlinger (*p* = 8.0 × 10^−85^) and Shifrut datasets (*p* = 2.2 × 10^−117^) when compared with models obtained from gene expression data (**Figure 6**). For specific mutations (TUBB gene, CDKN1B), the VIPER aggregation based on CCLE ARACNe networks seems to be particularly beneficial to increase the performance of mutation prediction models based on gene expression, while for a few mutations, such as RUNX1, the CCLE-based networks significantly decrease the model performance.

## Discussion

In this paper, we tested a framework to investigate the complex relationships between genetic events and transcriptional deregulation through machine learning approaches. We demonstrated as a generalized proof-of-principle that genomic alterations can be modeled by gene expression across several human cancers through several machine learning algorithms and, specifically, that a gbm approach seems optimal for the task. In the process, we generated a collection of models for each genomic alteration in each cancer context, showing that the best predicted alterations are not necessarily targeting known oncogenes or tumor suppressors. Interestingly, we show how the aggregation of gene expression profiles in groups of coexpressed genes, *via* the ARACNe/VIPER or WGCNA methods, makes the models more robust and more resistant to perturbations such as Gaussian noise or artificial down-sampling. Finally, we have shown how the same aggregation principle can have beneficial effects in predicting the presence of mutations in intrinsically noisy scenarios, both with artificial noise introduction and read reduction. At the same time, we have shown that expression-based mutation prediction can be modeled out in single-cell sequencing contexts, which can be considered as real cases of noisy datasets. The capability of predicting mutations based on scRNA-Seq is, however, reduced when compared with datasets derived from pooled cells sequencing, as those provided by the TCGA dataset: the average performances of TCGA models (**Figure 3**) generally rest on a range between 0.6 and 0.9 AUROC, while the performance of CROP-Seq models fall on an average value of 0.55 (**Figure 6**).

As transcriptional and signaling networks themselves gain diagnostic value, particularly for complex, multigenic diseases such as cancer (Alvarez et al., 2016), the network characteristics of coexpressed genes gain similar importance. A growing realization within the field of systems biology is that the activity and characteristic features of a given genomic network stem from the activity of smaller constituent subnetworks, and to this end, aggregated gene coexpression sets can constitute a novel and key focal point in network analysis overall (Wang et al., 2015).

The performance of gene aggregation methods has been tested before for sample clustering in RNA-Seq read reduction scenarios (Alvarez et al., 2016) but never in this specific task nor in a pan-cancer or a single-cell context. As a principle, the usage of robust averages of predefined coexpressed genes can be applied in any context where reliability of gene expression data is necessary, from differential expression to pathway enrichment analyses.

Using transcriptional networks with VIPER has been shown to be beneficial to increase the biological interpretability and reduce experimental noise in low-coverage sequencing setups such as the PLATE-Seq technique (Bush et al., 2017). We expect gene aggregation methods to further complement other RNA-seq noise reduction techniques (Ding et al., 2015), particularly those designed for scRNA-Seq data analysis. These include several recently published methods such as the deep count autoencoder (Eraslan et al., 2019), the factorial single-cell latent variable model (Buettner et al., 2017), the UnifiedRNA-Sequencing Model (Zhu et al., 2018), the single-cell Gene Expression Analysis app (Cai, 2019), the Ordering Effect gene Finder (Leng et al., 2016), and k-nearest neighbor smoothing (Wagner et al., 2017). Results obtained *via* computationally elegant techniques such as these stand to benefit from the inclusion of the types of network interaction features that we outlined previously.

Our analysis, while testing expression-based and network-based models for the entirety of frequent genomic alteration events in the TCGA dataset, is however limited to the presence/absence of single events considered separately. Patient tumor samples are often characterized by the co-occurrence of several mutations, CNVs, or a combination of those (Ciriello et al., 2013). In the future, generating models on a specific combination of genomic alterations will likely require larger clinical datasets, where each combination is represented in enough samples to allow for model training. This combinatorial approach for understanding the relationship between cancer genome and transcriptome will be beneficial in the context of personalized medicine, whereas every patient is considered separately (N-of-1 dataset), as it is characterized by a specific mutational landscape (Kristensen et al., 2014).

A recent study has shown, in agreement to our findings, that the highest part of cancer transcriptional variations are due to genomic alterations (copy number alterations and also somatic mutations) (Sharma et al., 2018) but also to epigenetic features and altered TF and µRNA balances. Those findings can explain why our results (**Figure 3**) highlight a highly variable performance depending on the modeled alterations and rare perfect models (max AUROCs rarely go above 0.9), while at the same time showing a generally better-than-random performance of expression-based prediction of genomic alterations (AUROC median and first quartiles >0.5). The notion that relationships between genomic alterations and gene expression profiles can be modeled across different cancer scenarios, as well as in single-cell and noisy contexts, may have important repercussions in diagnostics and quantification studies of heterogeneous cell populations, where theoretically a single quantitative expression experiment can be used to predict the presence or absence of a mutation.

## Materials and Methods

### Data Processing

We obtained raw expression counts, mutation, and CNV raw data from TCGA using the Firehose portal (gdac.broadinstitute.org). Raw counts were normalized using variance stabilizing transformation as described before (Giorgi et al., 2013). Somatic mutations not changing the amino acid sequence of the protein product were discarded. We flagged genes blacklisted by the MutSig project (Lawrence et al., 2013), such as TTN, ORs, MUCs as false positives, and removed them from further analysis (except the most mutated in the pan-cancer dataset, shown in **Figure S1**). CNV tracks were associated to the targeted gene using the GenomicRanges R package (Lawrence et al., 2013). Gene-centered CNVs were then associated to the expression profile of the gene itself. Genes affected by a CNV in more than 10 samples were used in the rest of the analysis. Samples with more than 0.5% of the genes in the genome somatically amplified, deleted, or mutated were deemed “hypermodified,” and the total number was shown in **Figure 1** bottom bars.

Clustering analysis was carried out on the TCGA tumor samples using the expression profiles of 1,172 TFs defined by gene ontology terms “transcription factor activity, sequence-specific DNA binding” (GO:0003700) and “nuclear location” (GO:0005634) (Ashburner et al., 2000).

The dataset expression profiles were visualized after TSNE transformation (van der Maaten and Hinton, 2008) with 1,000 iterations using a 2D kernel density estimate for coloring different tumor types (Duong, 2007). Oncogenes and tumor suppressor genes were obtained from the COSMIC Cancer Gene Census in October 2018 (Futreal et al., 2004).

### Modeling

We used the R *caret* package (Kuhn, 2008) v 6.0-81 as the platform to run all our predictive models in a standardized and reproducible way. Default parameters for model training were used. Binary classifiers were built to predict the presence/absence of mutation, amplification, and deletion events. The CNV value provided by TCGA corresponds to log2(tumor coverage) – genomic median coverage. The threshold for amplification/deletion presence was set to 0.5.

Data partitioning was performed once for each tumor type, with 75% of the samples used for training and 25% for test purposes. Training was performed using 10-fold cross-validation. Technical model robustness was assessed with a bootstrap approach as well (resampling of the patient samples with repetition). This was done in a smaller test scenario (bladder cancer mutation models) using the *caret* implementation of 100 bootstraps per mutation model (**Figure S10**). Bootstrap models have a slightly lower but not significantly different performance (AUROC Wilcoxon test *p* = 0.121) when compared with full dataset models. Recursive feature elimination was carried out by the default *caret* implementation on the 10,000 highest variance gene expression tracks. The algorithms used (and R packages implementing theme) were:

- Bayesian generalized linear model
- Tree models from genetic algorithms
- Gradient boost modeling (gbm)
- Generalized linear model
- k-nearest neighbors
- Linear discriminant analysis
- Neural networks
- Neural networks with feature extraction
- Random forest
- Linear support vector machine
- Radial support vector machine

In order to reduce information from the gene expression profiles, we adopted two strategies. The first, shown e.g. in **Figure 4B**, adds random Gaussian noise to the expression tracks, with a variable standard deviation (indicated as “Gaussian noise level”). Each model run after noise addition was run 100 times to allow for various data partitions. The second strategy (**Figure 5**) reduced the number of reads mapped to each gene in order to obtain expression samples with decreased total gene counts. In order to do so, we applied to each gene in each sample a down-sampling factor from a beta distribution:

$$\frac{1}{B\;(\alpha ,\;\beta )}x^{\alpha -1}{\left(1-x\right)}^{\beta -1}$$

where *B* is the beta function, acting as a normalization constant, *x* is the raw gene expression count in a particular sample, α is the first shape parameter, and β the second shape parameter. In order to reduce the total sample coverage to the desired level, β is set to 0.1 and α is set to:

$$\alpha =\frac{\beta f/r}{1-f/r}$$

where *f* is the desired number of reads and *r* is the total number of reads in the sample. A real case example of this beta distribution is shown in **Figure S11**.

### Aggregation Algorithms

We used ARACNe-AP (Lachmann et al., 2016) to generate TF-centered networks on each of the VST-normalized TCGA expression datasets. TFs were selected *via* gene ontology as described before, with *p*-value for each network edge set to 10^−8^. ARACNe networks were then used to obtain an aggregated value of TF activity for each sample using the VIPER algorithm (Alvarez et al., 2016) that reports the collective gene expression level changes of each TF-centered network vs. the mean expression of each gene in the dataset. Only TF networks with at least 10 genes (excluding the TF) were included.

WGCNA clusters of genes were constructed using the WGCNA package (Langfelder and Horvath, 2008) with default parameters and minimum network size set to 10. To obtain a robust median expression value for each WGCNA cluster in each sample, we used Tukey’s biweight function as implemented by the R *affy* package (Gautier et al., 2004).

### Single-Cell Analysis

We generated TF regulatory networks using ARACNe-AP as described before on the CCLE dataset available at https://portals.broadinstitute.org/ccle/data, raw counts version 2018-09-29, normalized by variance-stabilizing transformation (Pollen et al., 2014).

We downloaded raw RNA-Seq counts and guide RNA mutation data from single-cell CROP-Seq datasets, specifically: 1) the Datlinger dataset available on Gene Expression Omnibus (GEO) series GSE92872 (Datlinger et al., 2017), and 2) the Shifrut dataset was obtained from a healthy donor and is available as raw counts and cell-specific guide RNA from GEO sample GSM3375483 (Shifrut et al., 2018). Both single-cell CROP-Seq datasets were normalized using the R package Seurat with default parameters (Satija et al., 2015), as follows: a global-scaling normalization method (“LogNormalize”) was applied on raw gene counts for each cell; then, the values were multiplied by a scale factor (10,000 by default), and the results were log-normalized. These values were then regressed by two variables: unique molecular identifier counts and cell cycle, using cell cycle markers from (Tirosh et al., 2016). As an example of the Seurat regression, the TSNE representation of the Datlinger dataset before and after normalization clearly shows the removal of cell cycle bias effects (**Figure S12**).

Gradient boost modeling (gbm) was applied to each CROP-Seq dataset by aggregating cells carrying mutations on the same genes and using wild-type cells as control. Performance of gbm models using VIPER and expression variables was compared using a two-tailed Wilcoxon test on 100 repetitions of training/test set splits before cross-validation for model testing (Hanley and McNeil, 1982).

### Methods Availability

All code used to generate the analysis and the figures of this paper is available in the online materials as Supplementary Code.

## Data Availability

Publicly available datasets were analyzed in this study. This data can be found here: https://gdac.broadinstitute.org/

## Author Contributions

FG conceived the analysis. FG, FR and DM designed the analysis. FG performed the analysis. FG wrote the manuscript. FR provided scientific support on the VIPER algorithm. DM contributed to the single-cell analysis.

## Conflict of Interest Statement

The authors declare that the research was conducted in the absence of any commercial or financial relationships that could be construed as a potential conflict of interest.

## Supplementary Material

Figure S1 Table of most somatically mutated genes across TCGA tumor samples, in terms of number of samples where the gene is somatically mutated with altered protein product sequence. This table includes also MutSig-blacklisted genes (in gray) such as Titin (TTN), Obscurin (OBSCN), and Mucin genes.

Figure S2 Performance of 11 machine learning algorithms in binary classification of mutated/nonmutated samples using gene expression predictor variables in the liver hepatocellular carcinoma dataset. Each point corresponds to a specific mutation/model. Performance is indicated as AUROC: area under the receiver operating characteristic curve.

Figure S3 Relationship between alteration models and alteration frequency in the pan-cancer dataset, for mutations (left), amplifications (center), and deletions (right).

Figure S4 Performance of pan-cancer alterations models globally (left) and for MutSig genes, COSMIC oncogenes, and COSMIC tumor suppressors. The y-axis indicates rank-transformed AUROC values. Asterisks indicate a significant (<0.01) difference between a distribution and the global “other genes” distribution according to two-tailed Wilcoxon tests.

Figure S5 ROC curves for gbm TP53 models in breast cancer, using original expression data, VIPER aggregation (TF “activity”), and WGCNA aggregation (robust Tukey biweight average of clusters).

Figure S6 AUROCs of EGFR amplification gbm prediction models in glioblastoma with increasing noise, calculated using gene expression (black line) or aggregated gene expression using the WGCNA (green line) or VIPER (red line) algorithms.

Figure S7 AUROCs of EGFR amplification gbm prediction models in lung squamous carcinoma (LUSC) with increasing noise, calculated using gene expression (black line) or aggregated gene expression using the WGCNA (green line) or VIPER (red line) algorithms.

Figure S8 AUROCs of PVT1 amplification gbm prediction models in ovarian cancer with increasing noise, calculated using gene expression (black line) or aggregated gene expression using the WGCNA (green line) or VIPER (red line) algorithms.

Figure S9 AUROCs of PTEN deletion gbm prediction models in sarcoma with increasing noise, calculated using gene expression (black line) or aggregated gene expression using the WGCNA (green line) or VIPER (red line) algorithms.

Figure S10 Distribution of gbm models AUROCs for predicting bladder cancer mutations. Left: original models shown in the main study (Figures 2 and 3). Right: performance of models with bootstrap. The p-value of a two-tailed Wilcoxon test between the two distributions is indicated.

Figure S11 Beta distribution used to down-sample the 43.8M reads breast cancer sample TCGA-A1-A0SB-01 to 10M reads. The gray line shows the ratio between the target coverage and the original coverage.

Figure S12 TSNE representation of the Datlinger CROP-Seq dataset before (A) and after (B) removal of cell cycle-specific markers. Colors indicated the predicted cell cycle phase according to the Seurat pipeline [79].

Supplementary Table S1 AUROCs for each event in the pan-cancer TCGA dataset (24 tumor types with at least 100 samples with co-measured genomic and expression data. The sheet name indicates the tumor type and genomic alteration type (mut: somatic mutation, amp: amplification, del: deletion).

Supplementary Code R and bash code snippets used in this study.
