# Supplementary figures and images for: Pan-Cancer and Single-Cell Modeling of Genomic Alterations Through Gene Expression
Source: Front Genet. 2019 Jul 18;10:671. doi: 10.3389/fgene.2019.00671 (PMC6657420; doi:10.3389/fgene.2019.00671)

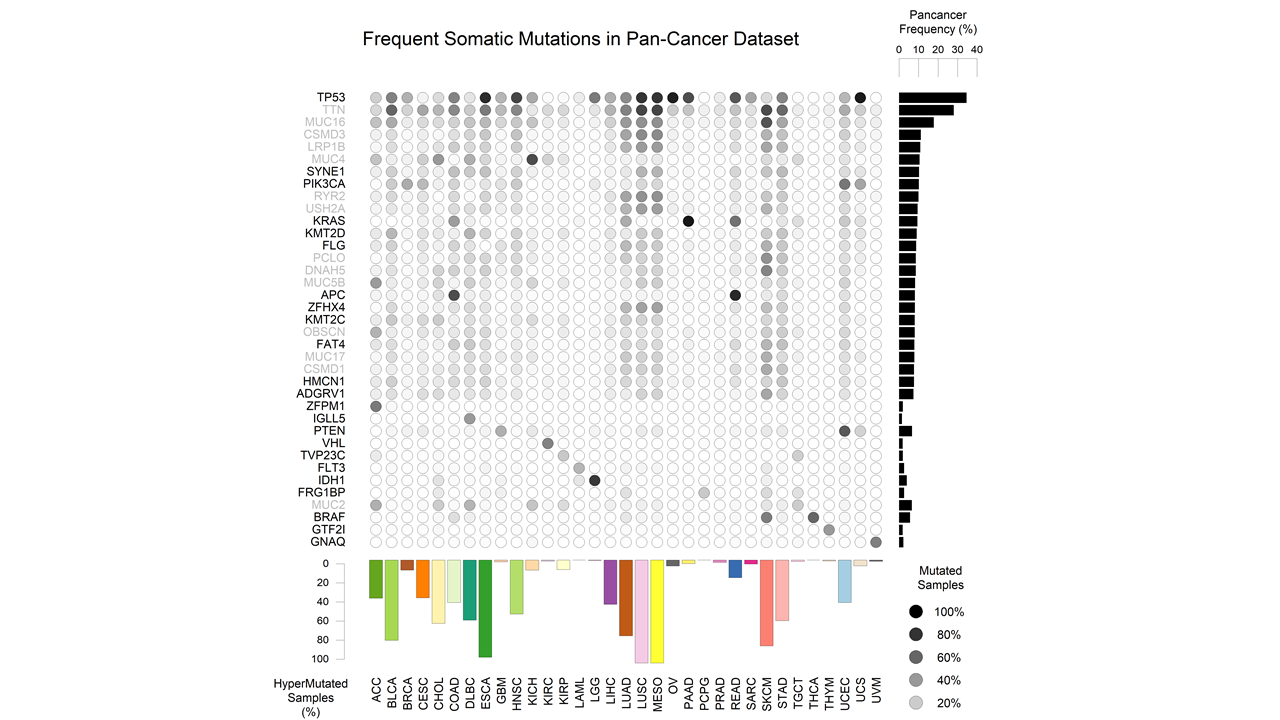

Supplement: Figure S1 — Table of most somatically mutated genes across TCGA tumor samples, in terms of number of samples where the gene is somatically mutated with altered protein product sequence. This table includes also MutSig-blacklisted genes (in gray) such as Titin (TTN), Obscurin (OBSCN), and Mucin genes. [file Image_1.tif]

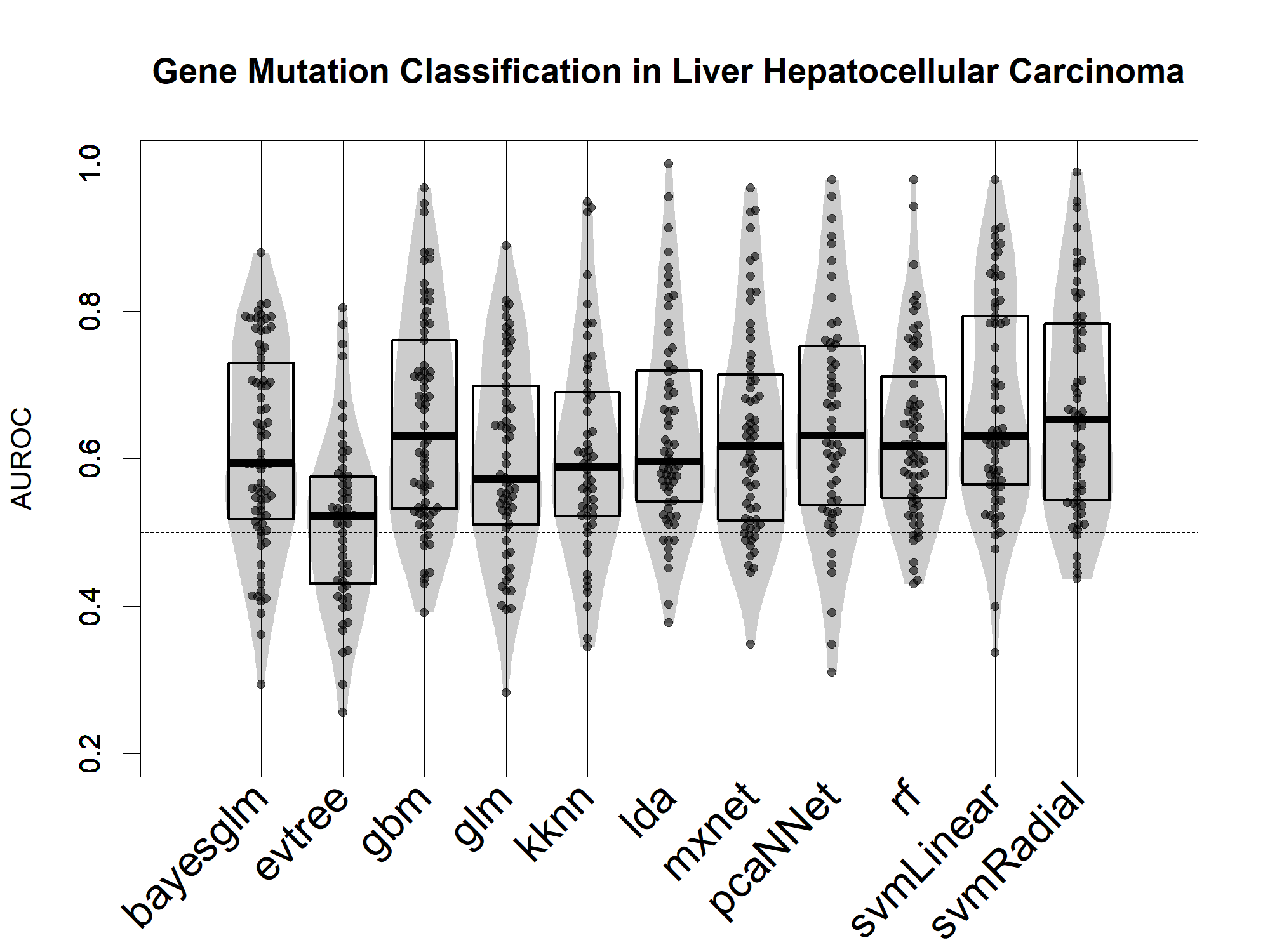

Supplement: Figure S2 — Performance of 11 machine learning algorithms in binary classification of mutated/nonmutated samples using gene expression predictor variables in the liver hepatocellular carcinoma dataset. Each point corresponds to a specific mutation/model. Performance is indicated as AUROC: area under the receiver operating characteristic curve. [file Image_2.png]

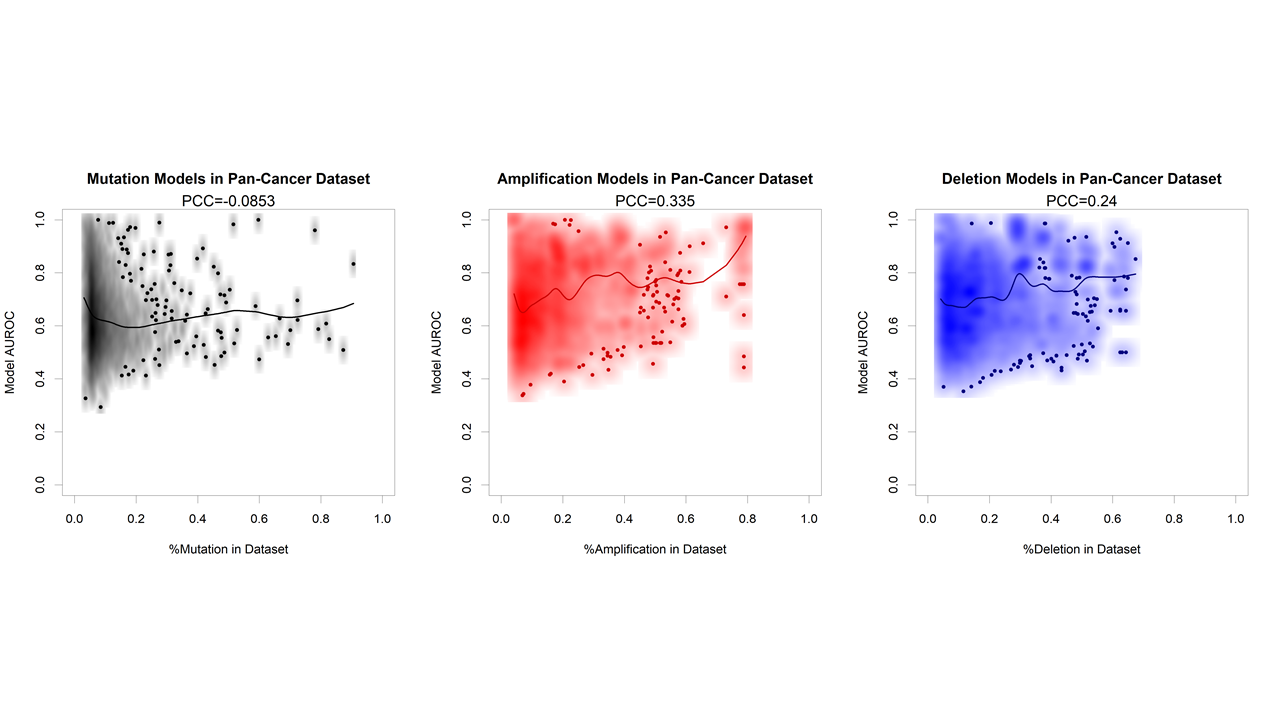

Supplement: Figure S3 — Relationship between alteration models and alteration frequency in the pan-cancer dataset, for mutations (left), amplifications (center), and deletions (right). [file Image_3.tif]

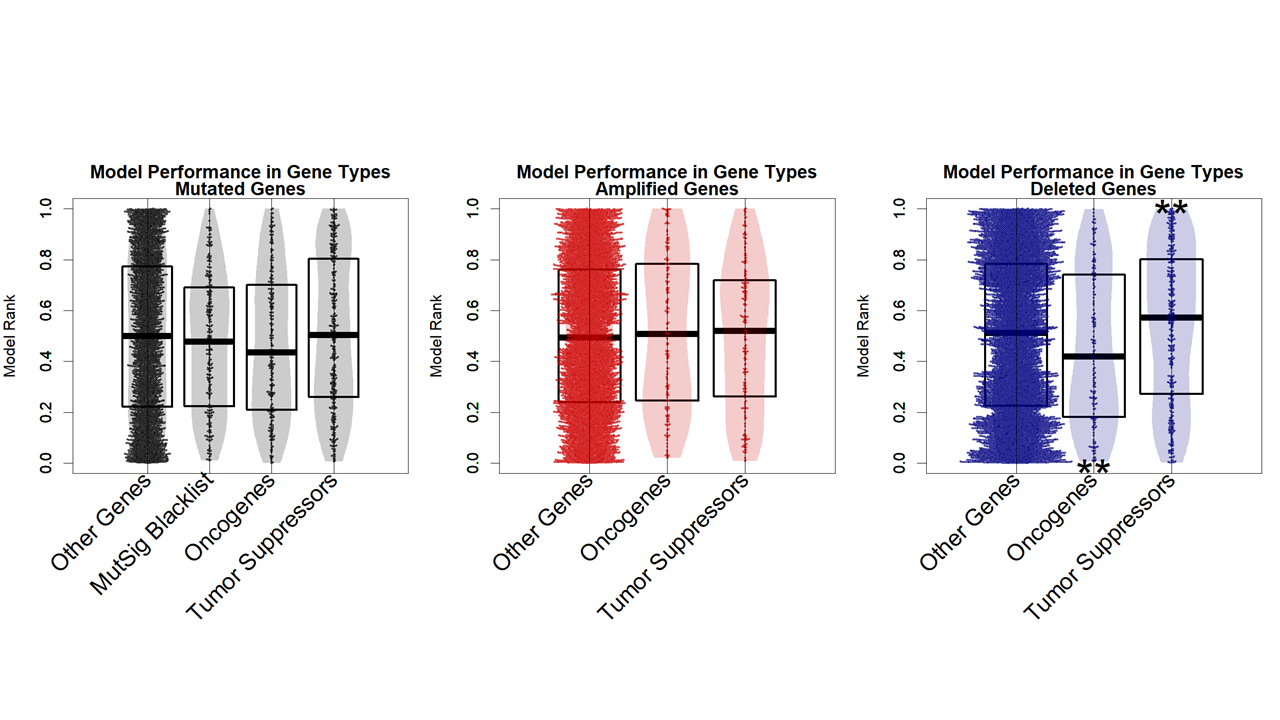

Supplement: Figure S4 — Performance of pan-cancer alterations models globally (left) and for MutSig genes, COSMIC oncogenes, and COSMIC tumor suppressors. The y-axis indicates rank-transformed AUROC values. Asterisks indicate a significant (<0.01) difference between a distribution and the global “other genes” distribution according to two-tailed Wilcoxon tests. [file Image_4.tif]

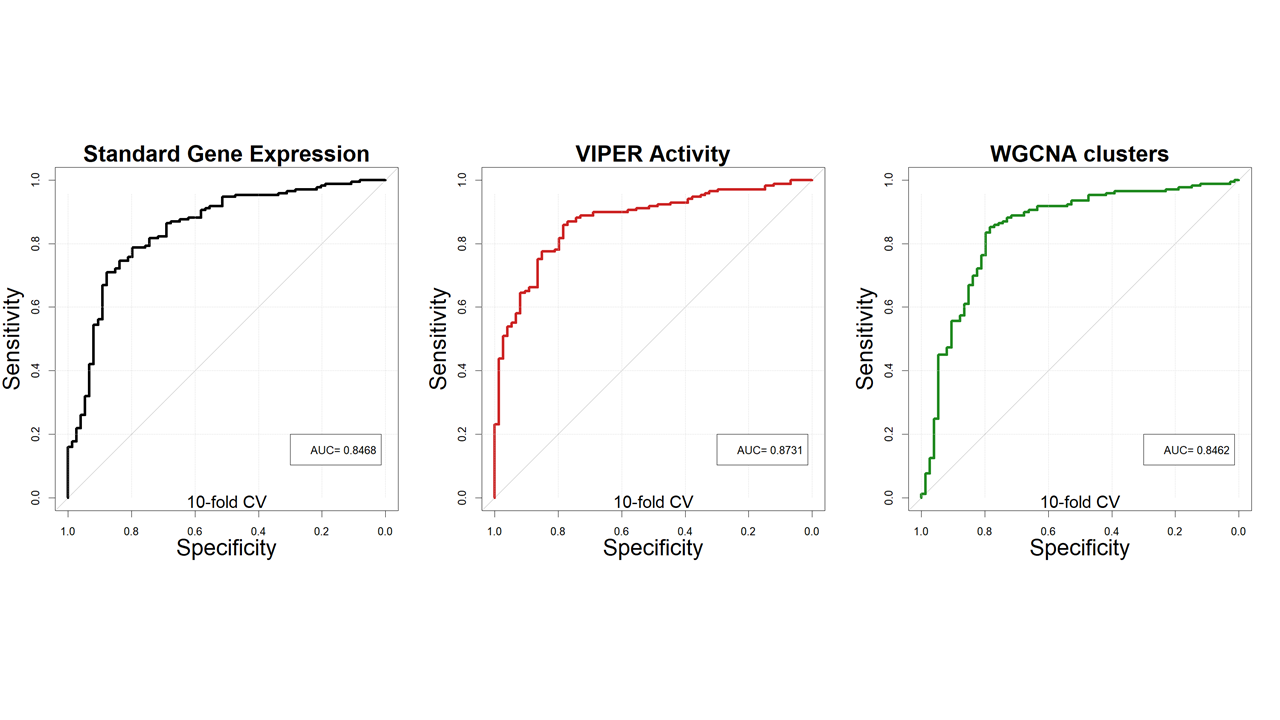

Supplement: Figure S5 — ROC curves for gbm TP53 models in breast cancer, using original expression data, VIPER aggregation (TF “activity”), and WGCNA aggregation (robust Tukey biweight average of clusters). [file Image_5.tif]

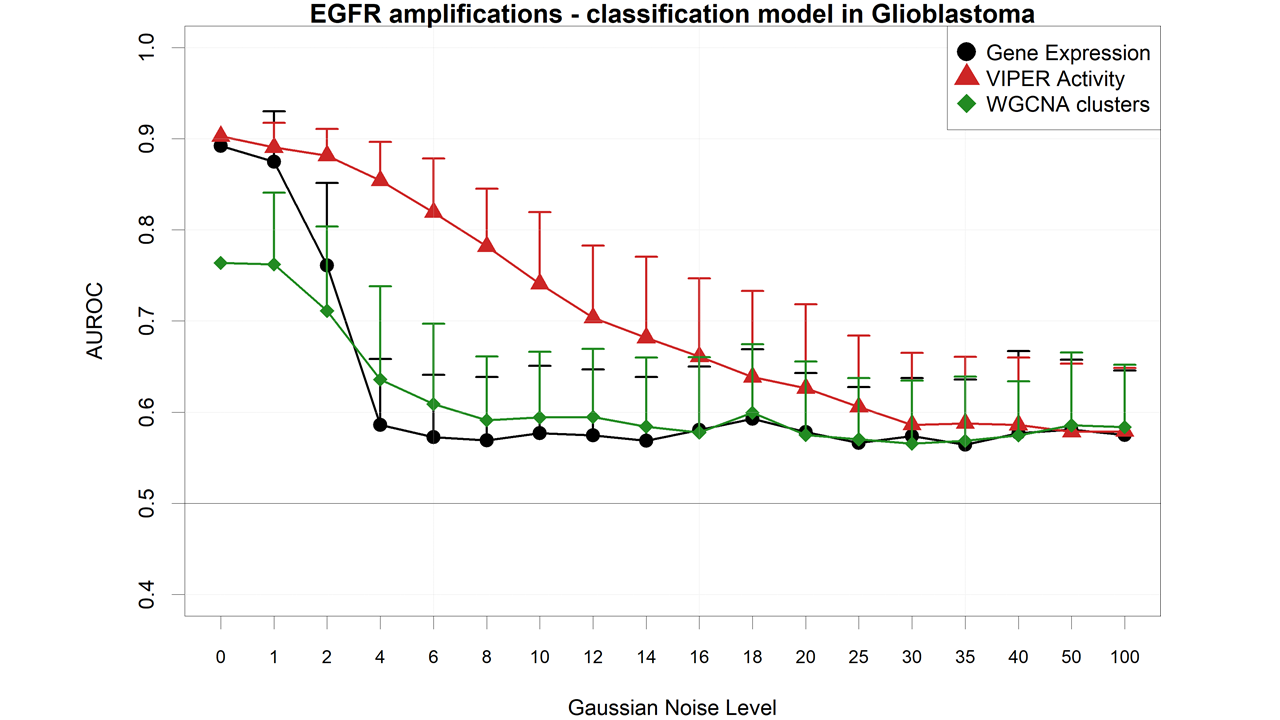

Supplement: Figure S6 — AUROCs of EGFR amplification gbm prediction models in glioblastoma with increasing noise, calculated using gene expression (black line) or aggregated gene expression using the WGCNA (green line) or VIPER (red line) algorithms. [file Image_6.tif]

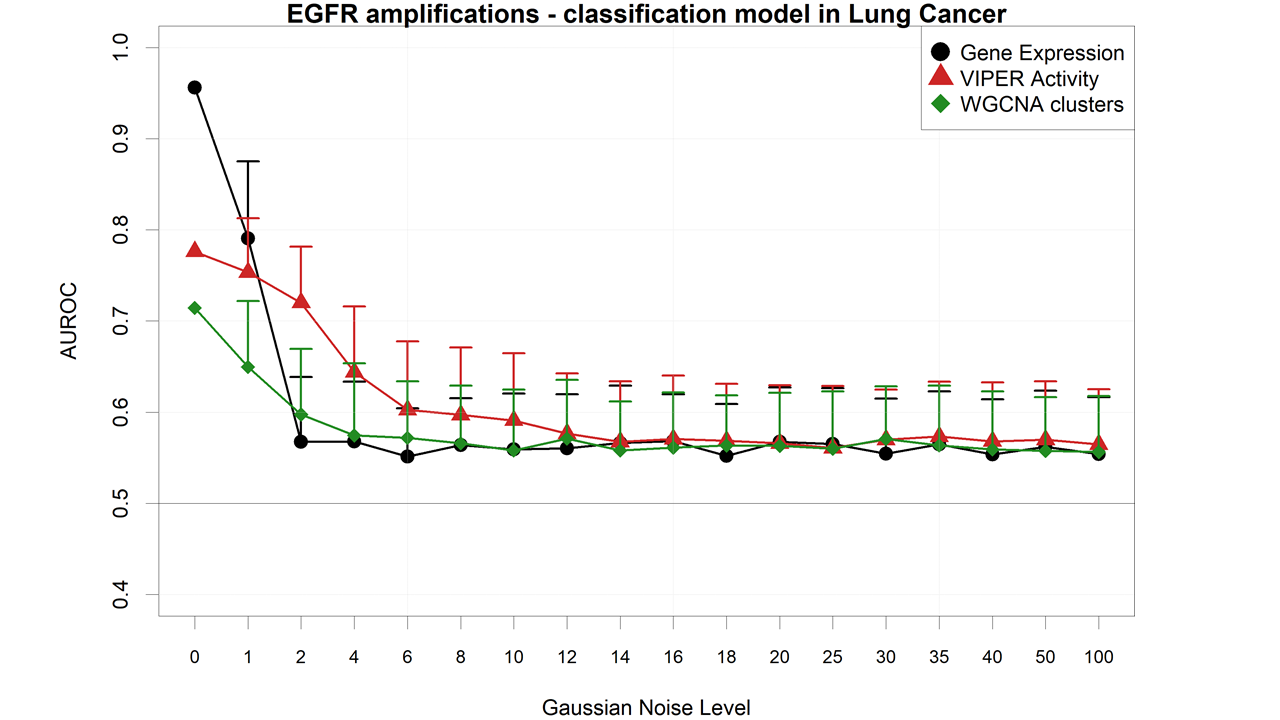

Supplement: Figure S7 — AUROCs of EGFR amplification gbm prediction models in lung squamous carcinoma (LUSC) with increasing noise, calculated using gene expression (black line) or aggregated gene expression using the WGCNA (green line) or VIPER (red line) algorithms. [file Image_7.tif]

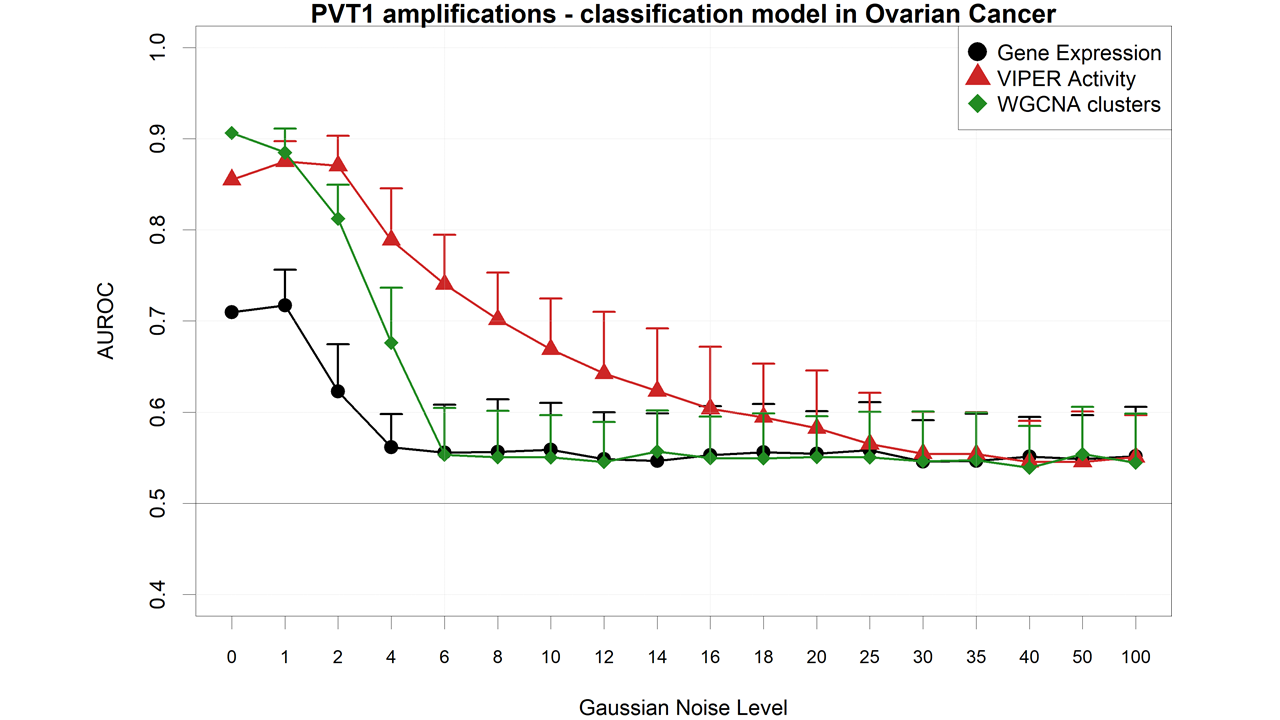

Supplement: Figure S8 — AUROCs of PVT1 amplification gbm prediction models in ovarian cancer with increasing noise, calculated using gene expression (black line) or aggregated gene expression using the WGCNA (green line) or VIPER (red line) algorithms. [file Image_8.tif]

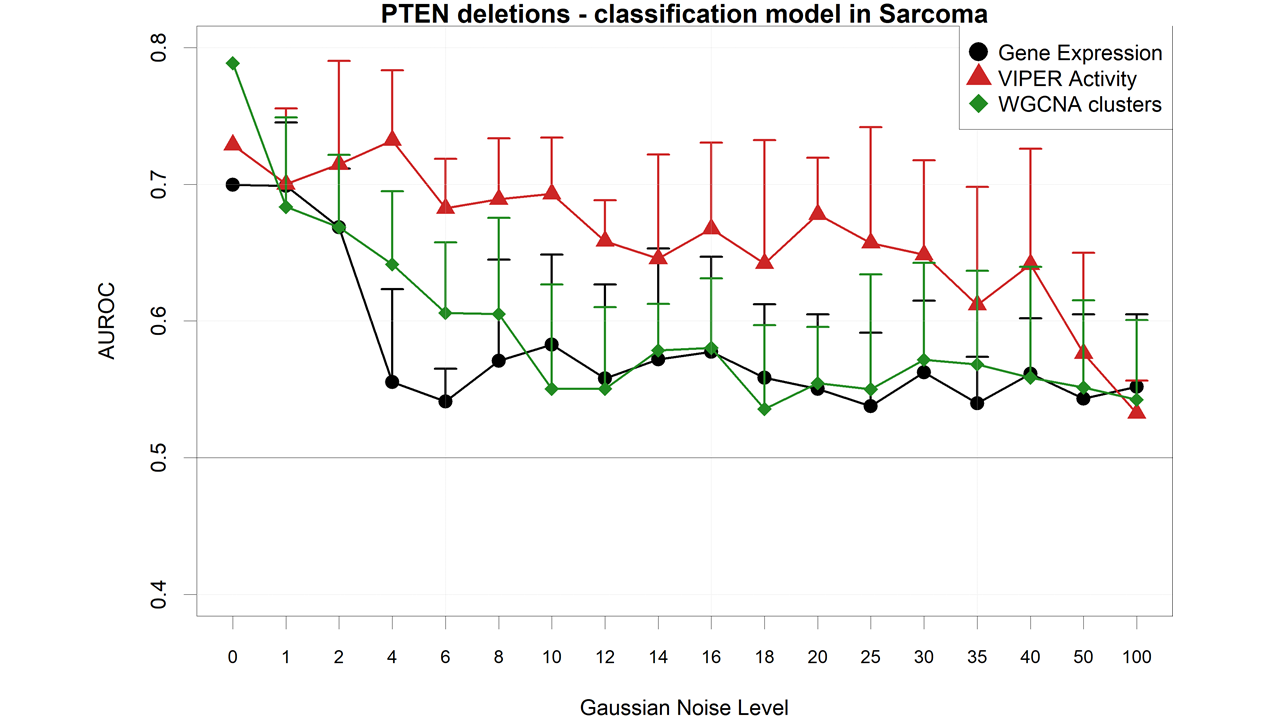

Supplement: Figure S9 — AUROCs of PTEN deletion gbm prediction models in sarcoma with increasing noise, calculated using gene expression (black line) or aggregated gene expression using the WGCNA (green line) or VIPER (red line) algorithms. [file Image_9.tif]

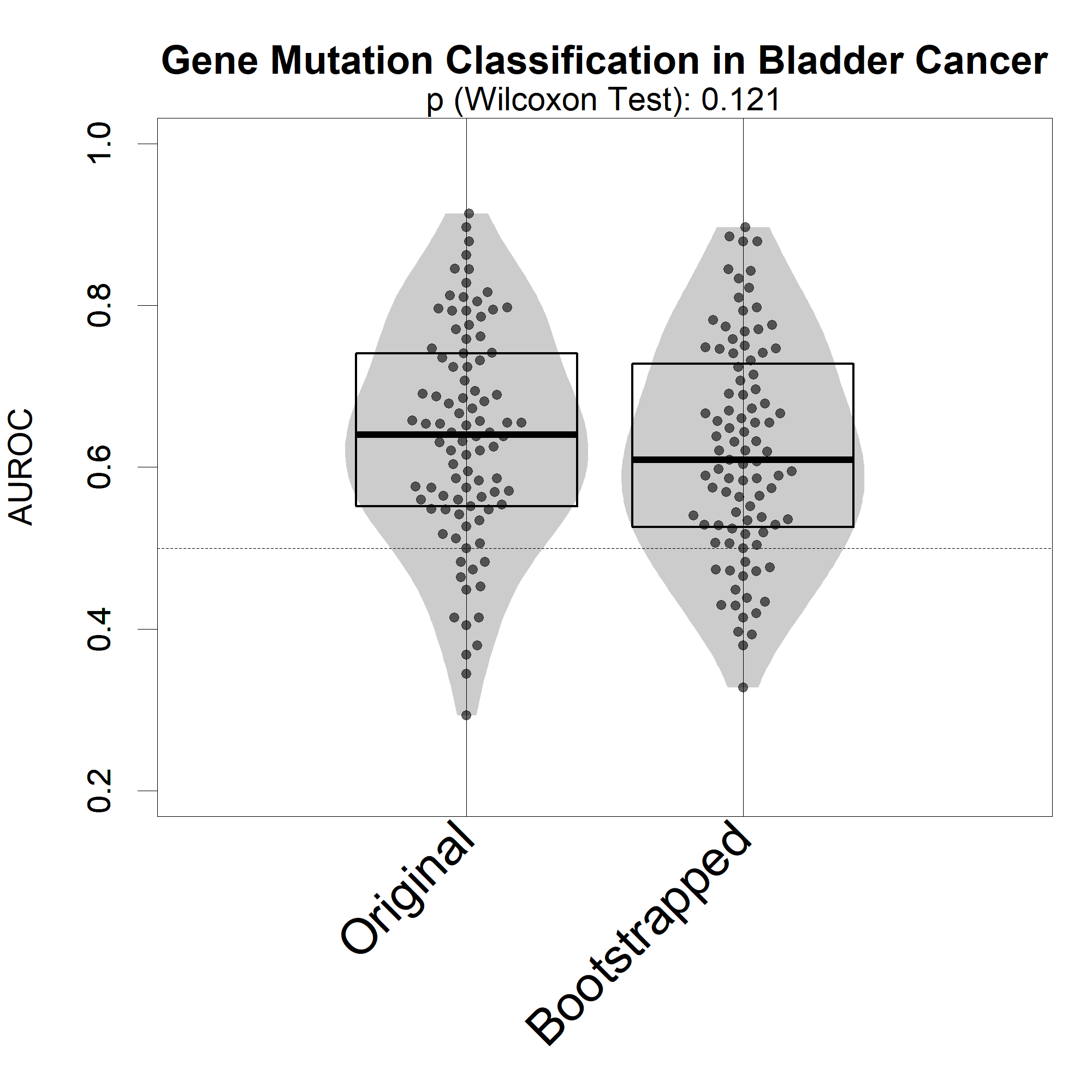

Supplement: Figure S10 — Distribution of gbm models AUROCs for predicting bladder cancer mutations. Left: original models shown in the main study (Figures 2 and 3). Right: performance of models with bootstrap. The p-value of a two-tailed Wilcoxon test between the two distributions is indicated. [file Image_10.png]

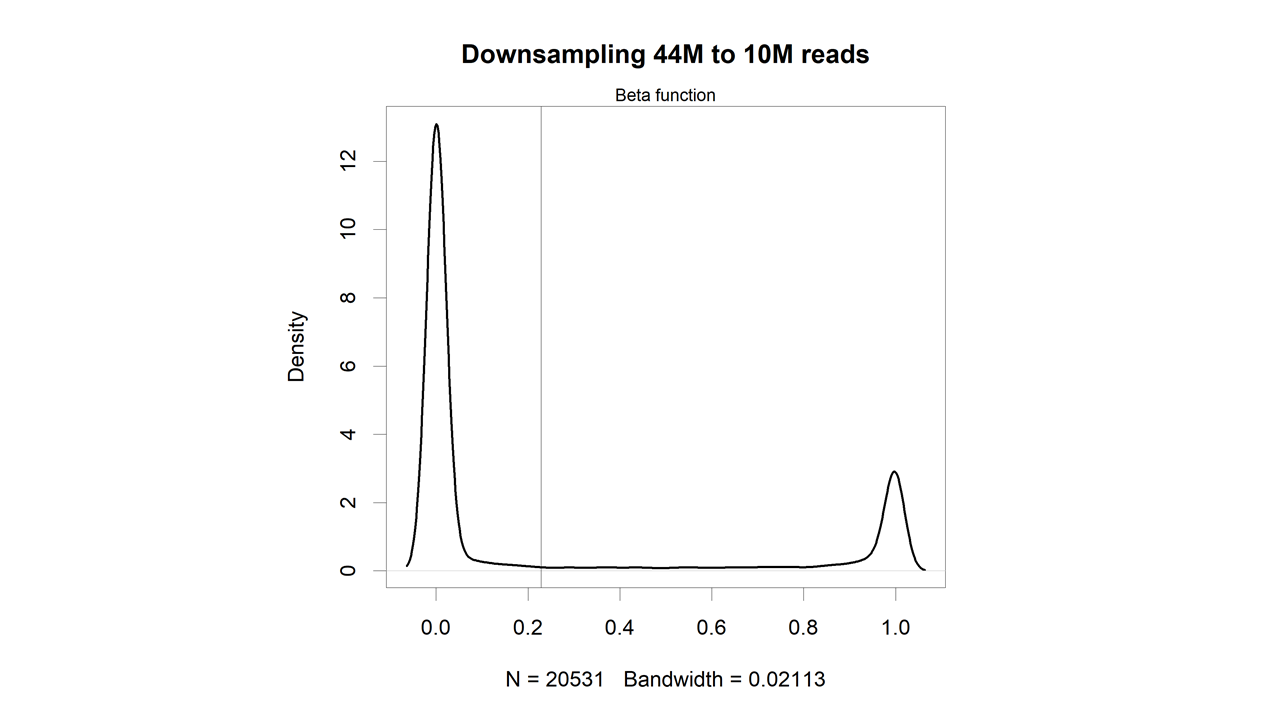

Supplement: Figure S11 — Beta distribution used to down-sample the 43.8M reads breast cancer sample TCGA-A1-A0SB-01 to 10M reads. The gray line shows the ratio between the target coverage and the original coverage. [file Image_11.tif]

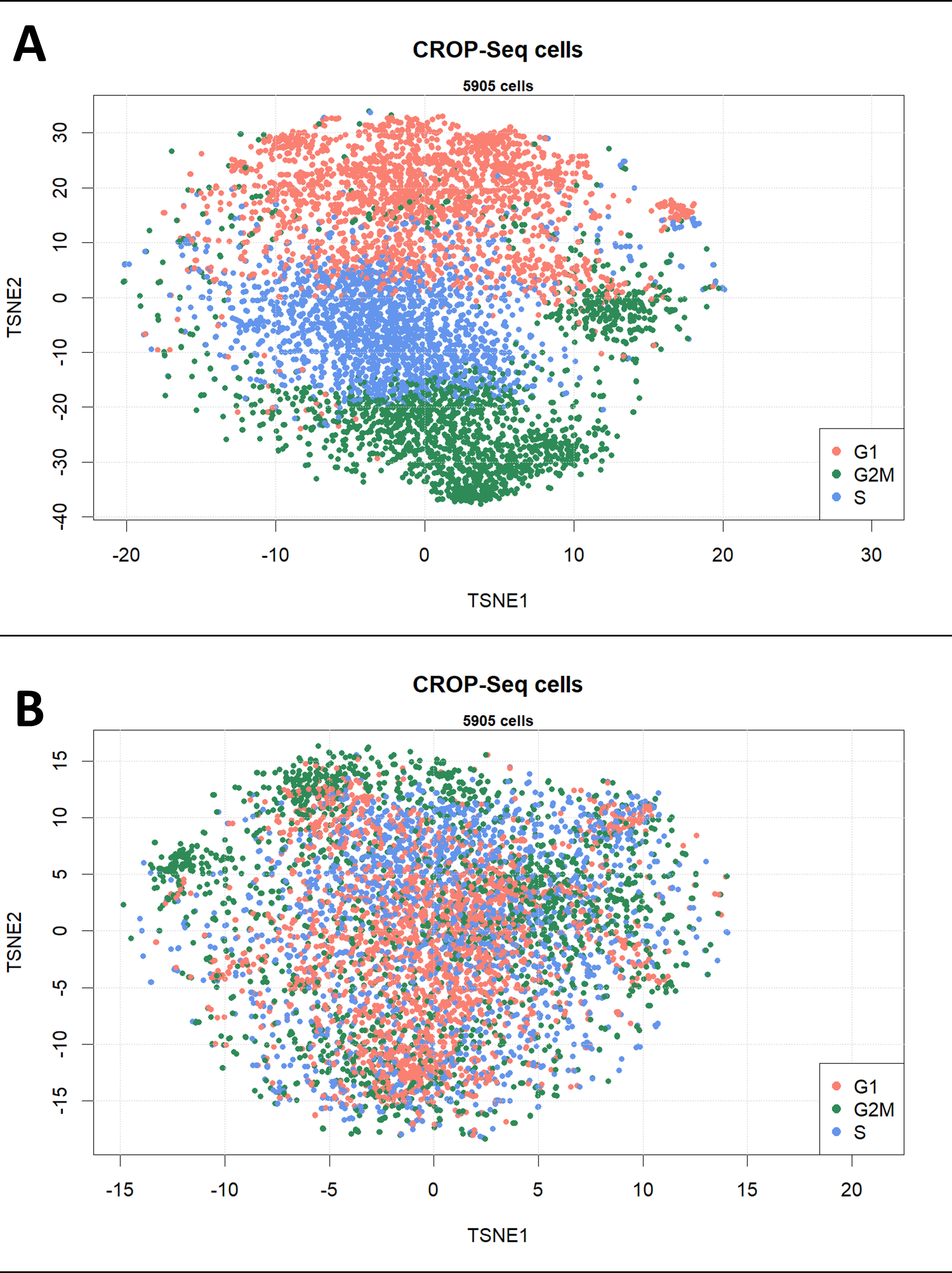

Supplement: Figure S12 — TSNE representation of the Datlinger CROP-Seq dataset before (A) and after (B) removal of cell cycle-specific markers. Colors indicated the predicted cell cycle phase according to the Seurat pipeline [79]. [file Image_12.tif]
